# Effect of 3D and 2D cell culture systems on trophoblast extracellular vesicle physico-chemical characteristics and potency

**DOI:** 10.3389/fcell.2024.1382552

**Published:** 2024-05-21

**Authors:** Norhayati Liaqat Ali Khan, Subhashini Muhandiram, Keerthie Dissanayake, Kasun Godakumara, Getnet Midekessa, Aneta Andronowska, Paul R. Heath, Suranga Kodithuwakku, Amber Rose Hart, Alireza Fazeli

**Affiliations:** ^1^ Division of Clinical Medicine, School of Medicine and Population Health, The Medical School, University of Sheffield, Sheffield, United Kingdom; ^2^ Centre of Preclinical Science Studies, Faculty of Dentistry, University Teknologi MARA (UiTM), Sg. Buloh, Selangor, Malaysia; ^3^ Institute of Veterinary Medicine and Animal Sciences, Estonian University of Life Sciences, Tartu, Estonia; ^4^ Department of Pathophysiology, Institute of Biomedicine and Translational Medicine, Faculty of Medicine, University of Tartu, Tartu, Estonia; ^5^ Department of Hormonal Action Mechanisms, Institute of Animal Reproduction and Food Research, Polish Academy of Sciences, Olsztyn, Poland; ^6^ Department of Animal Science, Faculty of Agriculture, University of Peradeniya, Peradeniya, Sri Lanka

**Keywords:** three dimensional, two dimensional, cell culture systems, extracellular vesicles, embryo implantation, potency

## Abstract

The growing understanding of the role of extracellular vesicles (EVs) in embryo-maternal communication has sparked considerable interest in their therapeutic potential within assisted reproductive technology, particularly in enhancing implantation success. However, the major obstacle remains the large-scale production of EVs, and there is still a gap in understanding how different culture systems affect the characteristics of the EVs. In the current study, trophoblast analogue human chorionic carcinoma cell line was cultivated in both conventional monolayer culture (2D) and as spheroids in suspension culture (3D) and how the cell growth environment affects the physical, biochemical and cellular signalling properties of EVs produced by them was studied. Interestingly, the 3D system was more active in secreting EVs compared to the 2D system, while no significant differences were observed in terms of morphology, size, and classical EV protein marker expression between EVs derived from the two culture systems. There were substantial differences in the proteomic cargo profile and cellular signalling potency of EVs derived from the two culture systems. Notably, 2D EVs were more potent in inducing a cellular response in endometrial epithelial cells (EECs) compared to 3D EVs. Therefore, it is essential to recognize that the biological activity of EVs depends not only on the cell of origin but also on the cellular microenvironment of the parent cell. In conclusion, caution is warranted when selecting an EV production platform, especially for assessing the functional and therapeutic potential of EVs through *in vitro* studies.

## 1 Introduction

Extracellular vesicles (EVs) have emerged as important mediators of intercellular communication in both humans and animals (Pitt et al., 2016; Gurunathan et al., 2022). The inherent properties of EVs in cellular communication hold great potential for diagnostic and therapeutic applications across a spectrum of diseases, including cancer (Bell et al., 2016), regenerative disorders (Ramasubramanian et al., 2019), cardio vascular (Chong et al., 2019), and infectious diseases (Kumar et al., 2020) etc. The growing recognition of the role of EVs in the process of embryo implantation has presented promising prospects for their potential utility in improving pregnancy rates in assisted reproduction technologies (ARTs) (Poh et al., 2023).

Embryo implantation is a highly co-ordinated process that initiates with molecular signalling from the embryo, followed by its apposition, attachment, and invasion in to the receptive uterine epithelium (Kim and Kim, 2017). Despite rapid advances in ARTs and reproductive medicine, the inability of the embryo to implant remains the bottleneck to successful pregnancy (Ma et al., 2023). Evidently, success of embryo implantation depends on three main factors, namely, the quality of the embryo (della Ragione et al., 2007; Ahlstrom et al., 2011; Oron et al., 2014), the endometrial receptivity (Achache and Revel, 2006) and the synchronized crosstalk between the two (Idelevich and Vilella, 2020). Despite the presence of a good quality embryo and an optimally receptive endometrium, inadequate communication between these two can result in pregnancy failure. This reciprocal communication between conceptus and endometrium is now widely believed to be facilitated by paracrine signalling (Guzeloglu-Kayisli et al., 2009; Yockey and Iwasaki, 2018). While soluble factors such as cytokines and growth factors have been critical in this interplay, the role of EVs is also starting to come to light (Godakumara et al., 2021; Hart et al., 2023; Muhandiram et al., 2023). EVs are a heterogeneous group of nano-sized particles (30–5,000 nm in size) secreted by almost all cell types into the extracellular environment. They play vital functions, including the maintainance of cellular homeostasis (Takahashi et al., 2017; Raposo and Stahl, 2019), and intercellular signalling in different physiological and pathological conditions. EVs carry a range of bioactive cargoes including proteins, lipids, and nucleic acids, to neighbouring or distant recipient cells (Raposo and Stahl, 2019; Cocks et al., 2021). EV molecular cargo is known to exert various functions within recipient cells. Recent studies propose that EVs secreted by the embryo might alter the uterine epithelium rendering it more receptive (Latifi et al., 2018; Kurian and Modi, 2019; Godakumara et al., 2021; Hart et al., 2022) and endometrial cell derived EVs in turn can affect the growth and development of the embryo (Mishra et al., 2021; Segura-Benítez et al., 2022; Aguilera et al., 2024). These unique characteristics of EVs hold great potential for both diagnostic and therapeutic applications in contemporary assisted reproductive practices, encompassing both human and livestock (Dissanayake et al., 2020).

Investigating the *in vivo* human embryo implantation process poses significant challenges due to the inaccessibility to the real *in vivo* environment and ethical concerns related to obtaining biological samples. The outer most layer of the pre-implanting embryo (blastocyst) consists with a layer of trophectoderm cells (responsible for blastocyst hatching, endometrial attachment and placentation) and the quality of these cells are known to correlate highly with the pregnancy progression in humans (Garcia-Belda et al., 2024). The EVs derived from trophoblast cells have shown to support endometrial receptivity and embryo attachment (Es-Haghi et al., 2019; Godakumara et al., 2021; Muhandiram et al., 2023). Trophoblastic EVs also play a role in facilitating epithelial-to-mesenchymal transition of endometrial cells and modulating inflammation during the pre-implantation period (by interacting with immune cells to mitigate maternal rejection of the embryo) (Shi et al., 2021). On the other hand, EV-encapsulation with specific molecules can be utilized to exert diverse effects in the endometrium (Taravat et al., 2023). Therefore, the characteristics of EVs derived from trophectoderm cells in their natural form or as engineered vesicles are of interest in investigating potential clinical interventions for implantation failure. Despite the substantial interest in EVs as a therapeutic and diagnostic modality, the large-scale generation of natural EVs remains a significant challenge (Syromiatnikova et al., 2022).

Cell lines are an attractive option for producing trophoblastic EVs on a large scale due to their ease of manipulation and ready availability. One of the most commonly utilized immortalized cell lines serving as an analogue of trophoblast cells is the human choriocarcinoma JAr cell line (Weber et al., 2021). JAr cells demonstrates significant similarities to human trophoblast cells in terms of hormone production and their morphology in general. The cell line is known to produce placental human chronic gonadodrophin (hCG), estrogen, and progesterone hormones in both monolayer and spheroid culture mimicking a pre-implanting embryo (White et al., 1988; King et al., 2000). For studies involving EVs, cells can be cultivated using three different culture techniques: 2D monolayer culture, 3D culture with scaffolds, and 3D culture without scaffolds (Langhans, 2018; Chen and Wang, 2020). The choice of cell culture method depends on the specific application. The trophectoderm of the pre-implantation embryo can be easily mimicked using simple spheroids generated from JAr cells (3D) in suspension culture (Ho et al., 2013; Weimar et al., 2013; Green et al., 2015; Li et al., 2017). In general, the scalability of 3D culture makes it easily adaptable for large-scale production of EVs as well. The 3D cultures are also known to better mimic cell-cell and cell-extracellular-matrix (ECM) interactions *in vivo* while replicating nutrient and waste disposal in cells in a spatio-temporal manner (Huh et al., 2011; Syromiatnikova et al., 2022). The main differences between 2D and 3D cell culture systems can be seen in cell shapes, nutrient distribution, formation of cellular junctions, cell proliferation rates, responsiveness to stimuli, and gene or protein expression profiles. Moreover, variations can occur in the culture duration and reproducibility (Vantangoli et al., 2015). These differences can affect the cellular functions as well. On the other hand, EVs also exhibit remarkable heterogeneity, with their molecular characteristics predominantly shaped by the originating cells, cellular microenvironment, and the metabolic state of the parent cells (Riazifar et al., 2017; Kalluri and LeBleu, 2020). Hence, it is critical to exam how the culture conditions (static or flow culture), fluid flow dynamics in suspension cultures, and the pattern of cell-matrix interactions affect the production of EVs. These factors may influence not only the cells themselves but also the EVs they produce, ultimately affecting their ability to modulate recipient cell functions. To study this, we first evaluated the impact of 2D and 3D cell culture micro-environment on the physico-chemical characteristics and proteome composition of EVs released by trophoblast cells.

However, there are no proper indicators to evaluate the functionality of trophoblastic EVs in embryo implantation. In a previous study, we demonstrated that increased secretion of Milk fat globule-EGF factor 8 protein (MFGE8 or human lactadherin) serves as a cellular response of EECs to trophoblast-derived EVs (Muhandiram et al., 2023), thereby functioning as a marker for EV signalling during embryo implantation. MFGE8 is a glycoprotein rich in cysteine (Wang, 2014). Interestingly, it displays changes in expression in the endometrium during the menstrual cycle and appears to be associated with endometrial receptivity (Bocca et al., 2012). MFGE8 in EECs is also known as required for embryo attachment to the endometrium demonstrating its functional significance in embryo implantation (Schmitz et al., 2014). Therefore, we used MFGE8 to examine the impact of 2D and 3D EVs on cellular signalling in EECs. Hence, in the current study we further evaluated the biological implications of JAr-EVs produced in 2D and 3D culture systems and their differences in function in an in vitro embryo implantation model.

## 2 Materials and methods

### 2.1 Cell culture and spheroid formation

The human choriocarcinoma cell line (JAr) and human endometrial adenocarcinoma cell line (RL95-2) were purchased from the American Type Culture Collection (HTB-144™, Teddington, United Kingdom). JAr cell line was originally established from the trophoblastic tumour of the placenta of a 24 year old woman and the cell line is from a male fetus (passage 55). JAr cells (HTB-144™, Teddington, UK) were cultured in T75 flasks with RPMI 1640 medium supplemented with 10% Fetal Bovine serum (FBS), 1% Penicillin Streptomycin (P/S) and 1% L-glutamine in 5% CO_2_ at 37°C. The media was changed every second day until the cells reached 80% confluency. For 2D monolayer culture, cells were harvested and 1  ×  10^6^ cells were cultured in 5 mL of supplemented RPMI 1640 medium in 60 mm petri dishes for 48 h. Next, the cells were washed with Dulbecco’s phosphate-buffered saline without Ca^+ 2^ and Mg^+ 2^ (DPBS, Verviers, Belgium) and changed to FBS free medium. Cell culture conditioned medium (CM) was collected after 6 h.

JAr spheroids were formed as described previously with some modifications (Es-Haghi et al., 2019). In brief, JAr cells at 80% confluency were washed with DPBS, harvested using trypsin-EDTA (Gibco^®^ Trypsin, New York, United States) and pelleted by centrifugation at 250 g for 5 min. Next, 1  ×  10^6^ cells were cultured in 5 mL of supplemented RPMI 1640 medium in 60 mm petri dishes on a gyratory shaker (Biosan PSU-2 T, Riga, Latvia), set at 295 rotations per minute (rpm) at 5% CO_2_ at 37 °C for 48 h to form spheroids (Supplementary Figure S1A). Then, spheroids were washed, replaced with FBS free media and CM was collected after 6 h. The spheroid viability was confirmed using Live/dead^®^ viability/cytotoxicity assay kit (Molecular Probes, Eugene, Oregon, United States), according to the manufacturer’s instructions (Supplementary Figure S1B).

RL95-2 cells were used as an analogue of receptive endometrial epithelial cells (EECs). Cells were maintained in Dulbecco’s Modified Eagles medium F12 (DMEM 12-604F, Lonza, Verviers, Belgium) supplemented with 10% FBS (Gibco™, 10500064), 1% P/S (Gibco™, 15140122, Bleiswijk, Netherlands), and 5 μg/mL insulin (human recombinant insulin, Gibco™, Invitrogen, Denmark) in 5% CO_2_ at 37°C.

### 2.2 EV isolation and characterization

EV isolation was carried out using methods described previously (Es-Haghi et al., 2019; Godakumara et al., 2021). EVs were harvested from CM collected from both 2D monolayer culture and 3D JAr spheroids (100 mL each). Soon after collection, CM was centrifuged at 400 *g* for 10 min. The resulting supernatant was further centrifuged at 4,000 g for 10 min and thereafter at 10,000 g for 10 min to remove cell debris and apoptotic bodies. Samples were then directly processed for EV isolation. CM was concentrated to 500 µL using Amicon^®^ Ultra-15 centrifugal filter devices (10 kDa cut-off). Next, EVs were purified using size exclusion chromatography (SEC). A gel filtration medium consisting of 4%–6% agarose matrix was used in 15 cm length columns to separate EV fractions from contaminating proteins. Fractions 7–10 were collected (each fraction was 500 µL in volume) and concentrated again to a total volume of 500 µL using a Amicon^®^ Ultra centrifugal filter device with a 10 kDa cut-off and stored in −80°C until further analysis. Characterisation of the isolated EVs was carried out using methods described in detail elsewhere (Es-Haghi et al., 2019; Midekessa et al., 2020; Godakumara et al., 2021). In summary, the nanoparticle size and concentration in EV fractions were measured using Nano Particle Tracking Analyser (NTA) (Particle Metrix GmbH, Inning am Ammersee, Germany). Transmission electron microscopy (TEM) was used for the physical characterization of EVs. Enrichment of EV protein markers was confirmed by label free proteomic analysis of 2D and 3D EVs. EVs derived from JAr 2D monolayer are referred to as 2D EVs. EVs from JAr spheroids are referred to as 3D EVs.

### 2.3 Fluorescence–nanoparticle tracking analysis (FL-NTA)

FL-NTA of 3D and 2D JAr EVs was carried out to measure the size and concentration of EVs following staining them with the flouroscent stain CMG (CellMask™ Green Plasma Membrane Staining, Thermo Fisher Scientific, Waltham, MA, United States), as described previously, with some modifications (Midekessa et al., 2021). In summary, JAr EVs purified in SEC were diluted separately in 1 × PBS to a particle concentration of about 1 × 10^10^ particles/mL. Before incubating EVs with CMG dye molecules, 1 µL of 5 mg/mL CMG stock (CellMask™ Green Plasma Membrane Staining, Thermo Fisher Scientific, Waltham, MA, United States) was added to 50 µL of PBS. Then, 1 µL of CMG in 1 × PBS was added to 10 µL diluted EVs and incubated at RT for an hour on a shaker at 350 rpm. All experimental tubes were covered with aluminium foil during incubation. After the incubation, the incubated samples were added to 990 µL of 1 × PBS suspension medium to achieve a final volume of 1 mL with a pH value of 7.2. The size and concentration profiles of both 2D and 3D JAr EVs were measured in the fluorescence mode using a ZetaView PMX 120 V4.1 instrument (Particle Metrix GmbH, Ammersee, Bavaria, Germany). Before the measurements, auto-alignment of the Instrument was performed using a known concentration of 100 nm polystyrene (PS) and fluorescent Yellow Green (YG) nanoparticles (Applied Microspheres B.V., Leusden, Utrecht, The Netherland). Subsequently, the particle concentration and size distribution were measured in triplicate with the following settings; Sensitivity:72, shutter: 100 and frames per cycle: 11. Number of particles were reported in both scatter mode (t-NPs) and florescent mode (Fl-NPs). PBS buffer-only and dye control were used to detect labelling artefacts before measurements.

### 2.4 Measurement of Zeta Potential (ZP)

EVs carry a net negative charge on the surface, and the ZP is a measure of the surface potential. Importantly, ZP is an indicator of colloidal stability and nanoparticle stability which are important parameters for EV pharmacokinetics. The zeta potential of EV preparations was measured using methods described previously with slight modifications (Midekessa et al., 2020, 2021). The zeta potential (ZP) of EVs was measured using ZetaView PMX 110 V3.0 instrument (Particle Metrix GmbH, Germany), three times at 25°C under the following scatter and fluorescent settings: Sensitivity was set at 72, shutter value at 100, and frame rate at 30 frames per second. For the size and ZP measurement of fluorescently labelled EVs, the sensitivity was set at 90. Data were analyzed by ZetaView NTA software. PBS buffer control was used to eliminate artefacts.

### 2.5 Sample preparation for protein quantification with liquid chromatography-tandem mass spectrometry (LC-MS/MS)

EV or conditioned media samples were precipitated with trichloroacetic acid deoxycholate (TCA-DOC) precipitation overnight. Approximate protein quantities were estimated based on the size of the pellets. The pellets were then solubilized in 7 M urea, 2 M thiourea, 100 mM ammonium bicarbonate (ABC), 20 mM methylamine buffer. Protein reduction was performed with 5 mM dithiothreitol (DTT) by incubating 1 h at room temperature. Protein alkylation was performed with 10 mM chloroacetamide by incubating 1 h at room temperature in the dark. Next, protease LysC (Wako) was added to an enzyme:substrate ratio (E:S) of 1:50, and the samples were incubated for 1 h at room temperature. Samples were then diluted five times with 100 mM ABC, trypsin (Sigma Aldrich) was added to 1:50 E:S ratio and incubated overnight at room temperature. After digestion, samples were acidified with trifluoroacetic acid (TFA) to a concentration of 1%, and samples were desalted using in-house made C18 StageTips. Samples were reconstituted in 0.5% TFA and peptide concentrations were determined with a Pierce colorimetric peptide assay (Thermo Fisher Scientific).

For MS analysis, 1 µg of EV peptides was injected to an Easy-nLC 1000 system (Thermo Scientific). The sample was eluted at 250 nL/min from the trap to a 75 µm ID ×50 cm emitter-column (New Objective) packed with C18 material (3 μm, 300 Å particles, Dr Maisch). The separating gradient was 2%–35% B 60 min and 40%–100% B 5 min [A: 0.1% formic acid (FA), B: 80% ACN + 0.1% FA]. Eluted peptides were sprayed onto a Q Exactive Plus (Thermo Fisher Scientific) quadrupole-orbitrap mass spectrometer (MS) using nano-electrospray ionization at 2.4 kV (applied through liquid-junction). The MS was operated with a top-5 data-dependent acquisition strategy. Briefly, one 350–1,400 m/z MS scan at a resolution setting of *R* = 70,000 at 200 m/z was followed by five higher-energy collisional dissociation fragmentation (normalized collision energy of 26) of five most intense ions (z: +2 to +6) at *R* = 17,500. MS and MS/MS ion target values were 3e6 and 5e4 with 50 m injection time. Dynamic exclusion was limited to 40 s.

### 2.6 Database searching and protein identification

Mass spectrometric raw files were processed using the MaxQuant software package (versions 1.6.15.0 and 2.0.3.0) to identify proteins with their respective label-free quantification values. Methionine oxidation, asparagine and glutamine deamidation and protein N-terminal acetylation were set as variable modifications, while cysteine carbamidomethylation was defined as a fixed modification. Label-free protein quantification (LFQ) was enabled with LFQ and protein minimum ratio count was set to 1. The search was performed against *Homo sapiens, Bos taurus* reference proteomes, using the tryptic digestion rule. Peptide-spectrum match and protein false discovery rate (FDR) were kept below 1% using a target-decoy approach. All other parameters were set to default. The mass spectrometry data are available in the ProteomeXchange Consortium via the PRIDE with the dataset identifier PXD048789.

Differential protein analysis on the identified proteins was carried out in LFQ-analyst (Shah et al., 2020) platform. Data were normalized based on the assumption that the majority of proteins do not change between conditions. In summary, contaminated proteins, proteins identified “only by site” and reverse sequences were filtered out. Proteins identified only by a single peptide and those not consistently identified/quantified in the same condition were also removed. The LFQ protein intensity values were log2 transformed, and missing values were imputed using “missing not at random” method. Protein-wise linear models, combined with empirical Bayes statistics were used for the differential expression analyses. A cutoff of the adjusted *p*-value of 0.05 (Benjamini-Hochberg method), along with a log2 fold change of 1 was applied to determine significantly up and downregulated proteins between 3D and 2D EVs. Differentially expressed proteins in the heatmap were clustered using k-means clustering.

### 2.7 Functional annotation and pathway enrichment analysis

Biological significance of differentially expressed protein list was determined by performing functional annotation, Gene Ontology (GO), and Kyoto Encyclopedia of Genes and Genomes (KEGG) pathway analysis using the Database for Annotation, Visualization and Integrated Discovery (DAVID) online platform (Huang et al., 2009; Sherman et al., 2022). DAVID determined the proportion of genes with a specific GO or KEGG functional annotation in the differentially expressed gene list relative to the proportion of those genes in the genome. All relevant differentially enriched gene IDs were submitted to the DAVID Bioinformatics platform for GO and KEGG pathway enrichment analysis. Gene set enrichment analysis of KEGG pathways were performed using the clusterProfiler package in R (Yu et al., 2012; Wu et al., 2021). A complete protein list with log2 fold change and FDR was submitted to clusterProfiler to perform gene set enrichment analysis and visualization. FDR significance cut off was ≤ 0.05.

### 2.8 MFGE8 enzyme linked immunosorbent assay

Cell culture supernatants (1 mL of media) were centrifuged at 400 *g* for 10 min to remove any contaminating cells, followed by centrifugation at 4000 *g* for 10 min and 10,000 *g* for 10 min to remove other cellular debris and apoptotic bodies. Samples were snap frozen in liquid nitrogen before storing in −80°C. MFGE8 protein concentration was measured in cell culture supernatants using a commercially available ELISA kit (Human MFGE8 Quantikine ELISA Kit, R&D systems) according to the manufacturer’s instructions. The optical density of each well was measured using a microplate reader set to 450 nm (Multiskan FC microplate photometer, Life Technologies, China).

### 2.9 Preparation of EV depleted cell culture media

EV depletion in FBS was carried out using methods described previously (Godakumara et al., 2021). FBS was filtered using Amicon ultra-15 centrifugal filters (100 kDa, MERCK KGAA, Darmstadt, Germany) at 3,000 g for 55 min and then used as a 10% supplementation to prepare RL95-2 cell culture media.

### 2.10 Statistical analysis

The size and concentration of EVs were expressed as mean ± SD. Statistical analysis was performed using One-way ANOVA (for multiple comparisons) or unpaired *t*-test. *p-*values < 0.05 were considered statistically significant marked with an asterisk (*) symbol. All the experiments were performed in three biological replicates.

### 2.11 Experimental design

#### 2.11.1 Determining the EV secretion from 2D vs 3D JAr culture

JAr cells were seeded in 1 × 10^6^ cells in 5 mL of supplemented RPMI 1640 medium in 60 mm petri dishes. Cells were kept on a gyratory shaker (Biosan PSU-2 T, Riga, Latvia), set at 295 rotations per minute (rpm) at 5% CO_2_ in 37°C for 48 h to form spheroids (3D culture). Similarly JAr cells were grown monolayer culture by growing 1 × 10^6^ cells in 5 mL of supplemented RPMI 1640 medium in 60 mm Petri dishes (2D culture) at 5% CO_2_ in 37°C for 48 h. AAfter 48 h, the JAr spheroids and monolayer cultures were washed with FBS-free complete medium. Next, the cell culture medium was replaced with FBS-free medium, and cells or spheroids were incubated for another 6 h before collecting the cell culture conditioned medium. Finally, cell culture supernatants were collected (100 mL each) and EVs were isolated. Particle concentration in EV samples were determined using nanoparticle tracking analysis.

#### 2.11.2 Determining the physico-chemical characteristics of the EVs derived from 2D vs 3D culture

EVs from JAr cells grown in 3D and 2D microenvironments were isolated using sequential centrifugation followed by SEC. Then the physical and biochemical characteristics (size, concentration, zeta potential) of the EVs were further analysed using NTA and LC-MS/MS. The protein cargo of 2D EVs was compared with 3D EVs using LC-MS/MS.

#### 2.11.3 Determining the MFGE8 secretion from receptive endometrial epithelial cells in response to 2D and 3D JAr EVs

MFGE8 secretion from EECs in response to 2D and 3D JAr EVs was determined using RL95-2 cells. The cells were seeded in 12 well plates (1 × 10^5^ cells/mL) and grown until they reached 85% confluency. After reaching the desired confluency, the cells were washed with DPBS, and EVs derived from 2D and 3D JAr cells were supplemented at a concentration of 1 × 10^9^ particles/mL in EV depleted medium and incubated for 24 h. Cell culture supernatants were collected at both 0 h and 24 h, and MFGE8 protein secretion from RL95-2 cells in response to 2D and 3D JAr EVs within 24 h was determined.

#### 2.11.4 Determining the functional implications of 2D and 3D JAr EVs

Finally protein cargo of 3D and 2D JAr EVs were compared with proteins secreted by human embryos (proteins in human blastocoels) prior to implantation (Poli et al., 2015) and proteins related to embryo implantation (Díaz-Gimeno et al., 2011; Enciso et al., 2018).

## 3 Results

### 3.1 Isolation and characterization of 3D and 2D EVs from human trophoblast cells

**FIGURE 1:**
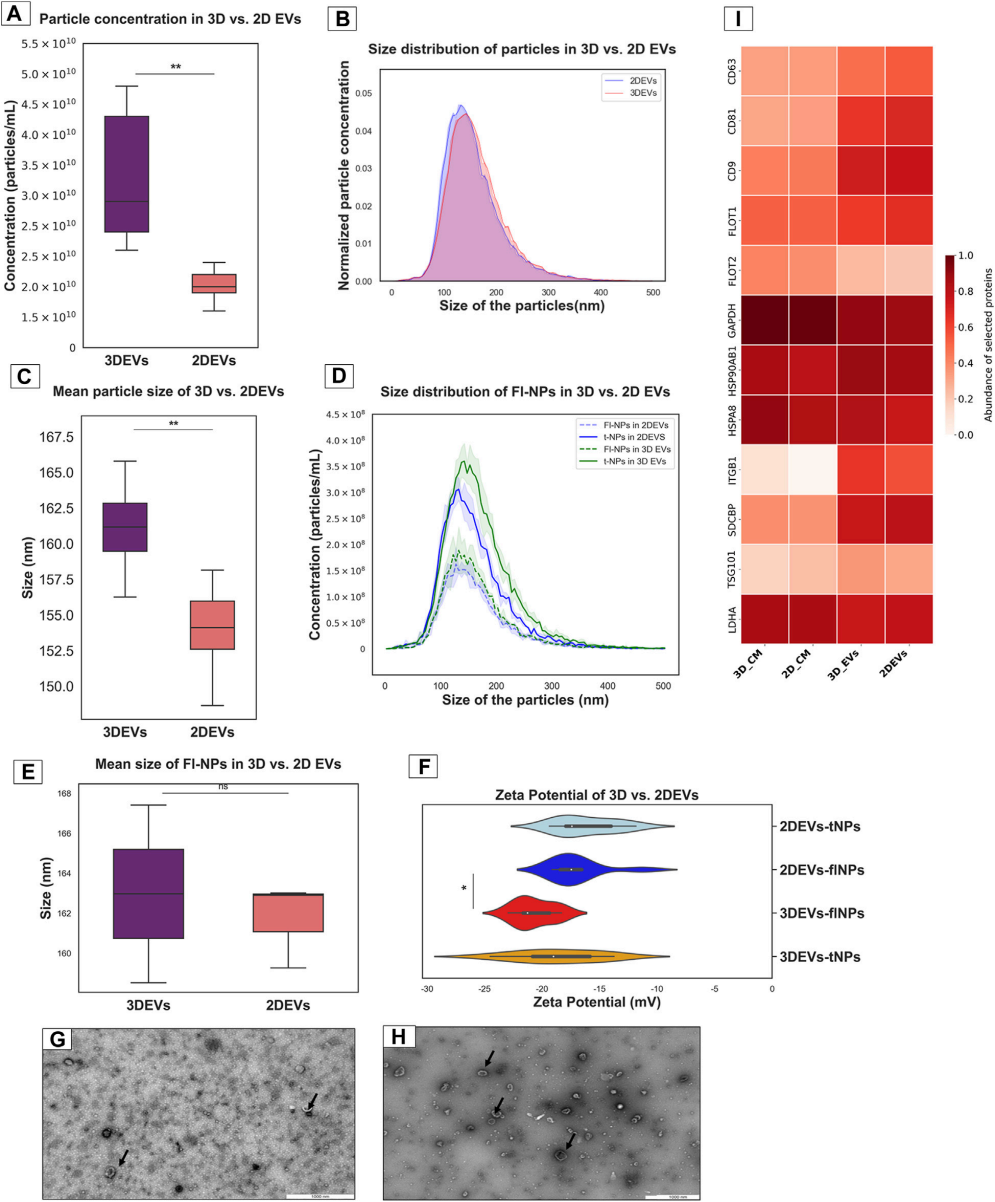
Characterization of nanoparticles derived from trophoblast cells grown in 3D and 2D cell culture systems as EVs **(A)**. Total particle count of nanoparticles released by 3D vs. 2D EVs **(B)**. The number and size profile of both 3D vs. 2D EVs exhibited a typical distribution of particles mainly less than 200 nm in size **(C)**. Mean size of nanoparticles released by 3D vs. 2D EVs **(D)**. Distribution of size and concentration of fluorescent nanoparticles released by 3D vs. 2D EVs **(E)**. Mean size of fluorescent nanoparticles released by 3D vs. 2D EVs. t-NPs: total nanoparticles, fl-NPs: florescent nanoparticles. **(F)**. Zeta potential of nanoparticles and fluorescently labelled nanoparticles in 3D vs. 2D EVs. tNPs: total nanoparticles, flNPs: florescent nanoparticles. EV morphology was assessed using TEM images of isolated EVs secreted from **(G)**. 2D and **(H)**. 3D cell culture systems. Scale bar: 1,000 nm. Black arrow shows EVs with typical characteristics **(I)**. The heat map illustrates the presence and enrichment of specific proteins in 2D EVs compared to 2D CM (2D_CM) and 3D EVs compared to 3D CM (3D_CM). The proteins reported in the heat map show enrichment of standard EV markers in 2D and 3D EV preparations. The mean LFQ value for each protein corresponding to each sample type was log transformed and re-scaled before visualization in the heat map. All the experiments were conducted in biological triplicate (*n* = 3), data were presented as mean ± SD, and *p* < 0.05 considered statistically significant.

EVs from 3D and 2D trophoblast cells were isolated and characterized using the methods described above. Total particle count was significantly higher in 3D EVs compared to 2D EVs (Figure 1A). The normalized EV size distribution graph illustrated that the majority of EVs produced by the two culture systems were predominantly in the 100–200 nm size range (Figure 1B). However, mean particle size was higher in 3D EVs compared to 2D EVs (Figure 1C). Next, membrane labelling of the nanoparticles was performed with CMG dye to characterize the EV population more specifically. The lipophilic membrane dye labelled a total of 55.81% of nanoparticles in the 3D EV samples and 53.77% in the 2D EV samples, respectively (Figure 1D). However, the average size of the labelled particles did not exhibit significant differences between 3D and 2D EVs (Figure 1E). The zeta potential of the 3D and 2D sample nanoparticles were similar. Interestingly, the zeta potential of the fluorescently labelled nanoparticles in 3D vs. 2D EVs was significantly different with 3D EVs having a more negative zeta potential (Figure 1F). TEM analysis demonstrated that both 3D and 2D nanoparticles were less than 200 nm in diameter and uniform in nature with typical EV characteristics such as dual membrane, cup shape, and circular cross section (Figures 1G,H). Mass spectrometry analysis of EV samples and their respective conditioned media samples showed that classical EV protein markers such as CD9, CD81, CD63, FLOT1, and TSG101 were enriched in both 3D and 2D EVs samples compared to their respective CM samples (Supplementary Table S1). Enrichment of EV protein markers in 3D and 2D EVs and CM samples were semi quantitatively visualized in the heat map (Figure 1I). In total 11 EV protein markers and 1 purity marker (LDHA) were shown in the current analysis, confirming that our EV samples are enriched with the majority of known EV proteins. LDHA did not show significant enrichment, suggesting the EV preparations mainly consists of EVs, with limited non EV protein contaminations. EV protein markers were selected according to the five-component framework of MISEV guidelines for reporting protein composition of EVs (Welsh et al., 2024).

### 3.2 Proteomic composition of 3D vs 2D EVs

**FIGURE 2:**
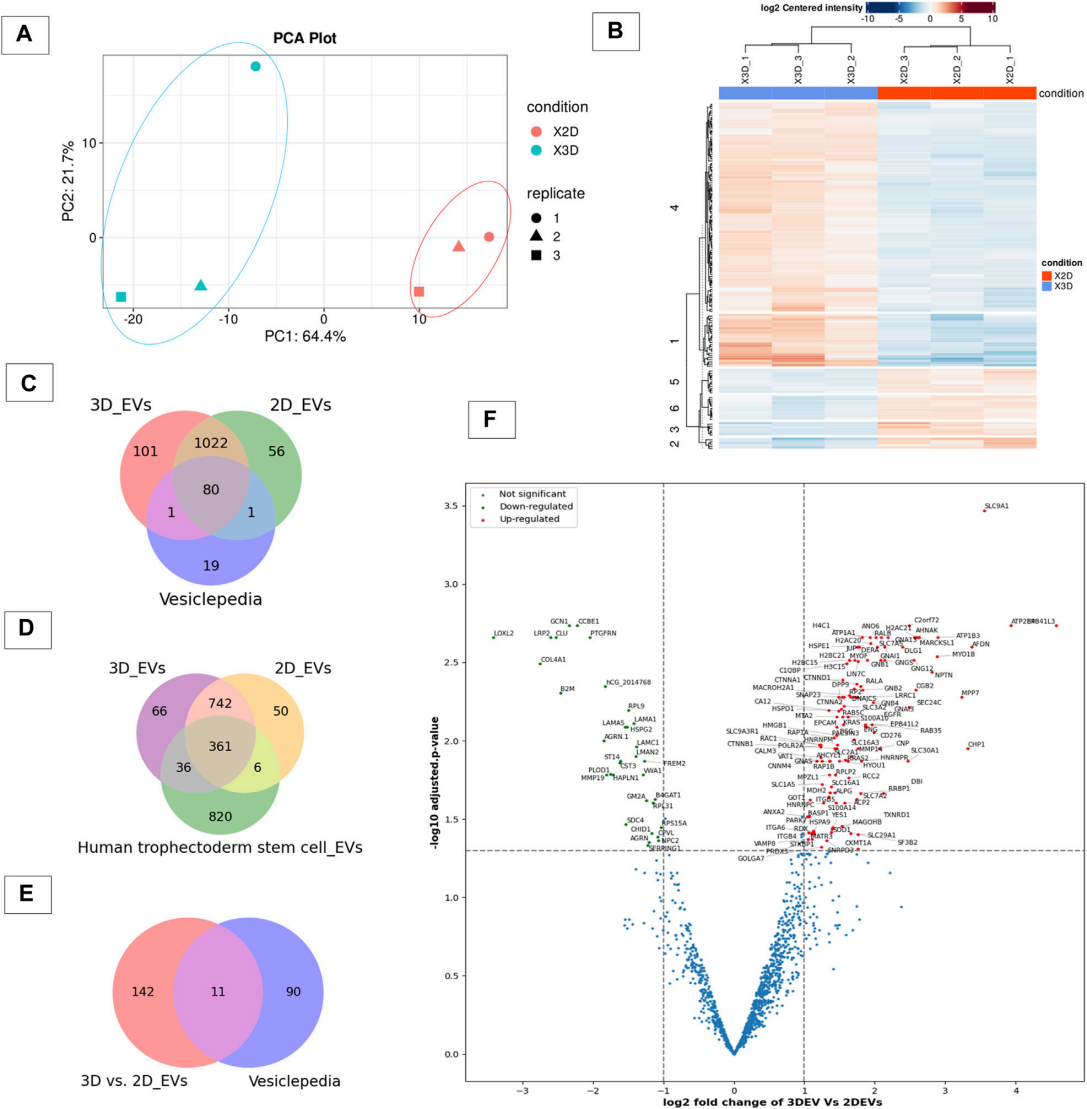
Proteomic profile of 3D vs 2D EVs. **(A)** Principle component analysis of protein composition of 3D vs. 2D EVs showing distinctive proteomic profiles. **(B)** Heat map of clustering among samples based on differentially enriched proteins between 3D and 2D EVs. **(C)** Venn diagram displaying the number of proteins identified in EVs derived from cells grown in 3D and 2D conditions and vesiclepedia most common proteins (top 100 proteins). **(D)** Venn diagram displaying the number of proteins identified in EVs derived from JAr cells grown in 3D and 2D conditions and proteins enriched in human trophectoderm stem cell derived EVs (Poh et al., 2021). **(E)** Venn diagram of EV proteins differentially expressed between 3D and 2D culture conditions and vesiclepedia top most common proteins (top 100 proteins). **(F)** Volcano plot showing differential expressed proteins between 3D and 2D EVs.

To gain insight in to whether the cell culture growing conditions affected the protein expression in 3D and 2D cell derived EVs, we analysed their protein cargo profile using label-free mass spectrometry. A total of 1,313 proteins were confidently detected with FDR< 0.01 in at least one of the three replicates of the 3D and 2D EV groups, where 1,204 proteins were detected in 3D EV and 1,159 proteins in 2D EV group, respectively. The principle component analysis and hierarchical clustering separated EVs derived from specific cell types, indicating global proteomic changes based on cell culture conditions (Figures 2A,B). Total of 1102 proteins were found to be common to both EV types. Interestingly, 102 proteins were uniquely detected in 3D EVs, whereas 57 proteins were uniquely detected in the 2D EV group. Of the 1,102 proteins that were common to both EV types, 80 were common with the top 100 EV proteins reported in Vesiclepedia database (Figure 2C). The 3D and 2D EV proteomic profile shared significant similarity with previously reported proteins profile of human trophectoderm stem cell derived EVs as well (Figure 2D). The commonly shared proteins included antioxidants (PRDX2, PRDX6), adhesion molecules (ITGB1, ITGB5) and cytoskeleton regulators (RAC1 and RHOA) etc. These proteins can play roles in antioxidant defences, cell adhesion and signal transduction processes which are considered important in embryo implantation (Poh et al., 2021). Differential expression analysis of the protein composition between 3D and 2D EVs resulted in 153 differentially expressed proteins (DEP). Of the 153 proteins (Supplementary Table S2), 120 proteins were upregulated, and 33 proteins downregulated. Among the 153 protein changes, 11 proteins are known to be classical EV proteins according to vesiclepedia (ANXA2, GNB1, SLC3A2, BSG, GNAS, RAB5C, RAC1, GNB2, ATP1A1, RAP1B, and RALA) (Figures 2E,F).

**FIGURE 3:**
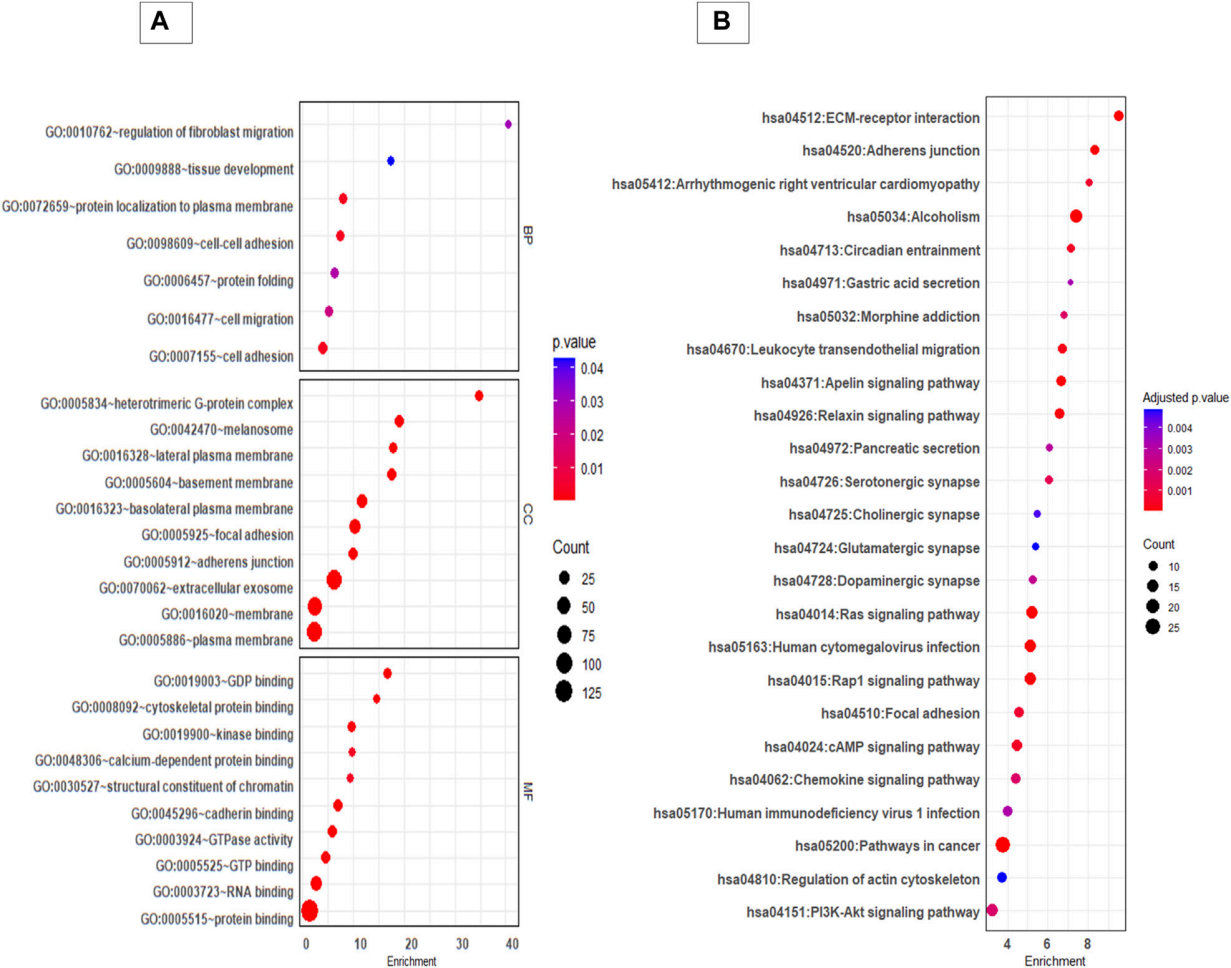
GO and KEGG pathway analysis of differentially expressed proteins between 3D and 2D EVs. **(A)** GO analysis of biological pathways (BP), cellular components (CC) and molecular function (MF). **(B)** KEGG enrichment pathways of differentially expressed proteins between 3D and 2D EVs. The colour of the bubble chart show the adjusted p. value and gene count in each term is represented by bubble size.

GO and KEGG pathway analysis provided insight in to the biological background of the DEP (Figures 3A,B). The significantly enriched (FDR< 0.05) GO biological terms (BP) revealed that cell-cell adhesion, protein localization to plasma membrane, and cell migration were enriched in EVs from 3D cells compared to 2D cells. For molecular function (MF), pathways such as GDP binding, cytoskeleton protein binding, and cadherin binding were enriched. The differentially expressed protein list was mapped to the KEGG database, and it was found that the identified proteins were enriched in 25 pathways (FDR< 0.05). Taken as a whole, pathways like ECM receptor interaction, adherens junctions, Ras signalling pathway, focal adhesion, regulation of actin cytoskeleton, and P13K-Akt signalling pathways were highly enriched in 3D EVs compared to 2D EVs (Supplementary Tables S3, S4).

**FIGURE 4:**
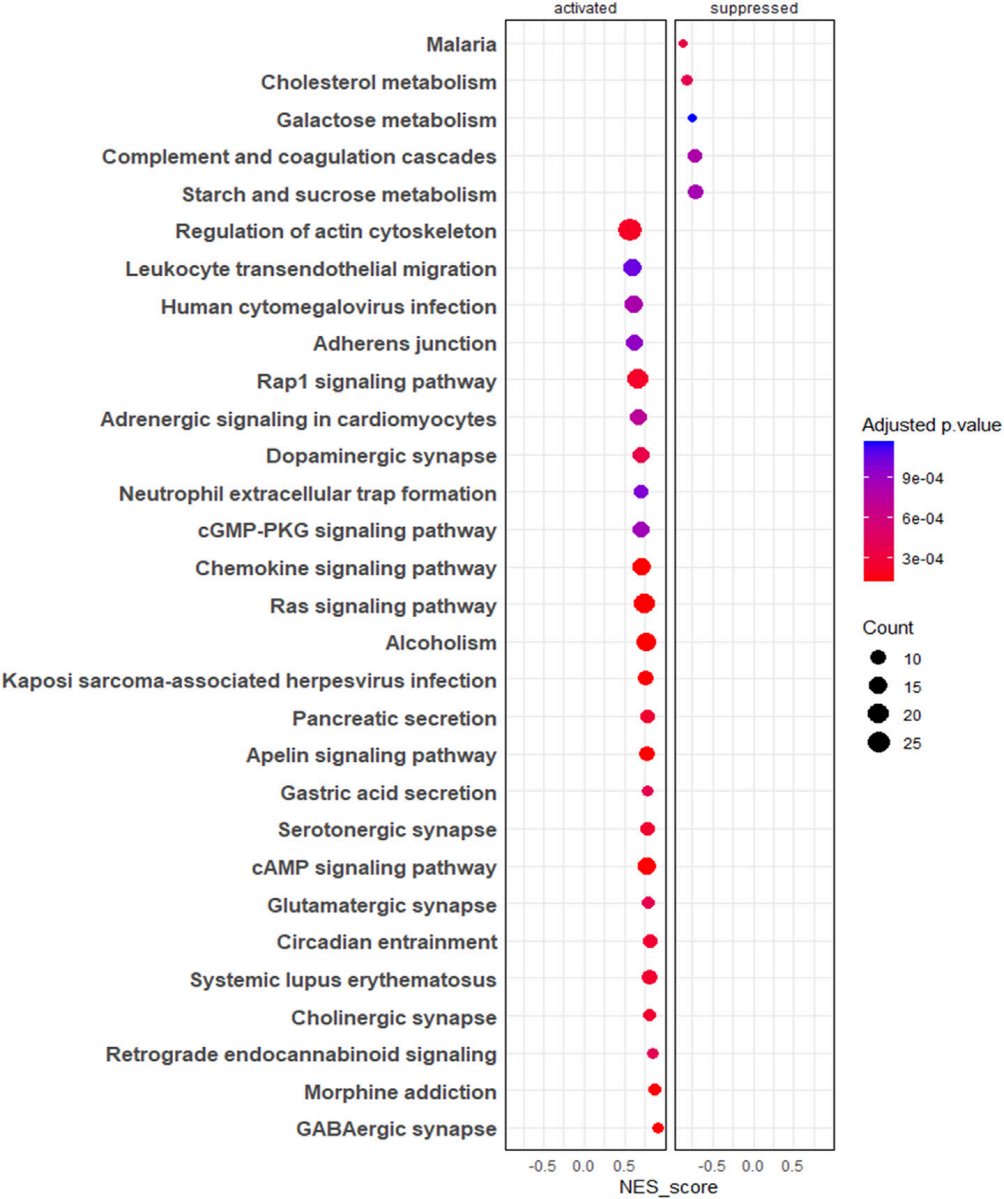
Gene set enrichment analysis (GSEA) of KEGG pathways. The colour of the bubble chart show the adjusted *p*. value and gene count in each term is represented by bubble size.

The gene set enrichment analysis of KEGG pathways (Supplementary Table S5) revealed pathways such as regulation of actin cytoskeleton, adherent junctions, Ras signalling and Rap signalling were profoundly upregulated in 3D EVs compared to 2D EVs, whereas pathways like cholesterol metabolism and galactose metabolism were downregulated (Figure 4).

### 3.3 Endometrial epithelial cells sense the trophoblastic EV cargo changes

**FIGURE 5:**
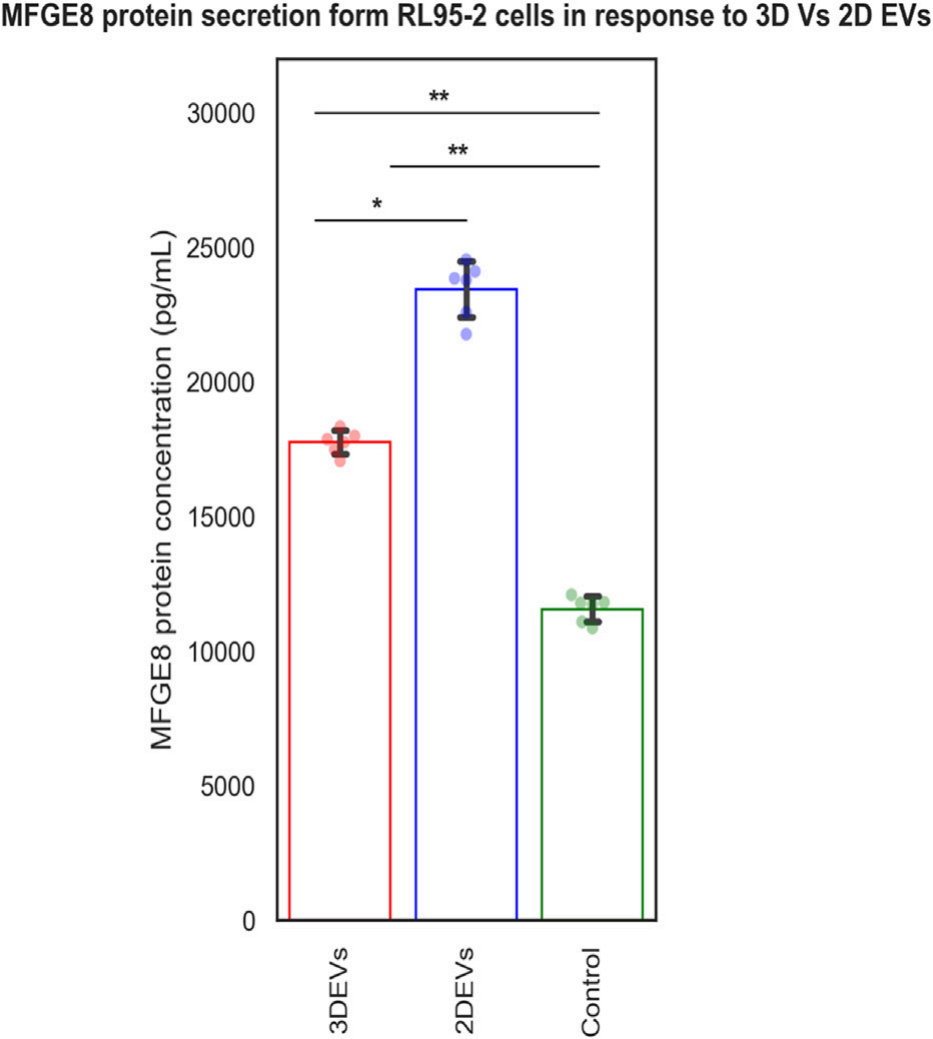
MFGE8 secretion from RL95-2 cells in response to 3D vs 2D EVs.

The MFGE8 secretion was increased in both 3D and 2D EV treated RL95-2 cells compared to its control. Increased MFGE8 secretion was seen in 2D EV treated RL95-2 cells compared to 3D EVs suggesting that 3D and 2D EVs have different potencies in trophoblast EV mediated cellular signalling (Figure 5).

### 3.4 Trophoblast cell derived extracellular vesicles may have the potential to prepare endometrium for embryo implantation

**FIGURE 6:**
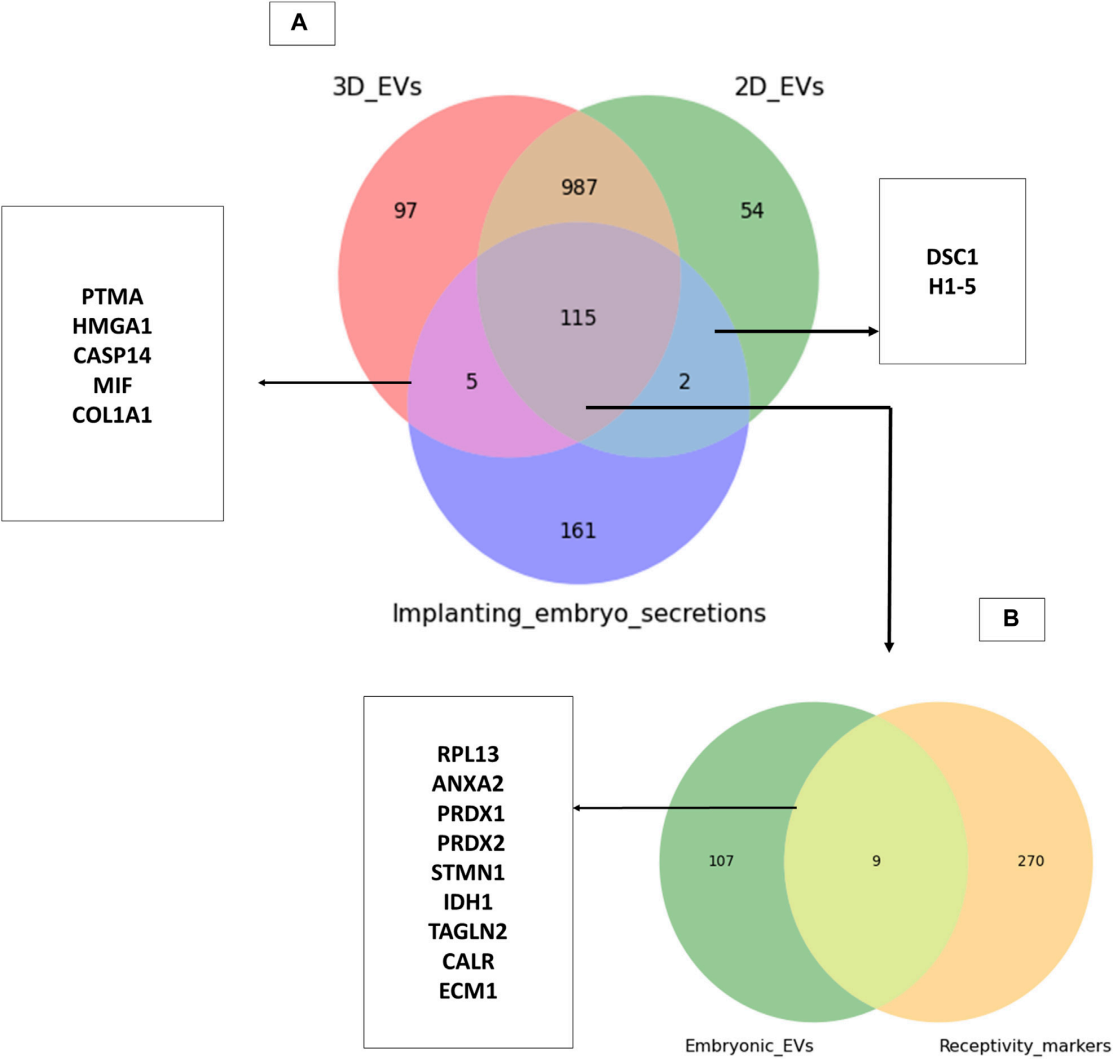
Common proteins between trophoblastic EVs and human embryo secretions. **(A)** Comparison of 3D and 2D trophoblast cell derived EVs with secretions of human embryos prior to implantation (Poli et al., 2015). **(B)** Comparison of proteins common to trophoblast EVs and human embryo secretions to known endometrial receptivity markers (Díaz-Gimeno et al., 2011; Enciso et al., 2018).

Trophoblasts cells are critical cellular players of embryo implantation. Here, we investigated whether the trophoblast analogue JAr cell derived EVs resemble the embryo secretions in humans (blastocoel proteome from embryos produced for *in vitro* fertilization). We identified around 115 proteins that are commonly found within embryo secretions and EVs secreted by trophoblastic cells (Supplementary Table S6). However, a total of 120 proteins were shared between 3D EVs and embryo secretions, whereas 117 proteins were shared between 2D EVs and embryo secretions. (Figure 6A). Among them, 115 proteins common to 2D EVs, 3D EVs and human embryo secretions. Some of these proteins were known to play roles in endometrial receptivity (ANXA2, CALR, STMN1) (Cheng et al., 2009; Gou et al., 2015; Yoshie et al., 2021), antioxidant defence (PRDX1, PRDX2) (Wu et al., 2017), trophoblast attachment and invasion by actin polymerization (TAGLN2) (Liang et al., 2019) etc. (Figure 6B).

Through manual searching of the list of proteins (115) that were common to both trophoblast EVs and human embryo secretions, we discovered additional proteins that may play a role in the process of embryo implantation (Table 1). The common proteins for EVs and embryo secretions have been indicated in the process of embryo implantation and hence may have a therapeutic value in preparing the endometrium for embryo implantation.

**TABLE 1:** Proteins commonly shared among pre-implanting single embryo secretions, 3D and 2D trophoblast EVs and their known role in the process of embryo implantation.

Protein symbol	Protein name	Implications in embryo implantation, embryo development and endometrial receptivity	References
S100A14	S100 calcium binding protein A14	Highly enriched in trophectoderm and have a role in trophoblast invasion during embryo implantation	Munch et al. (2016)
LDHA	Lactate dehydrogenase A	Maintain trophoblast cell cycle and proliferation via PI3K/AKT/FOXO1/CyclinD1 pathway	Gardner, 2015; Zhu et al. (2023)
PGK1	Phosphoglycerate kinase 1	Plays a role in endometrial decidualization	Tong et al. (2018), Long et al. (2023)
G6PD	Glucose-6-phosphate dehydrogenase	G6PD deficiency induced disruption of redox balance leads to diminished NADPH and elevated lipid peroxidation in embryo causing embryo lethality	Nicol et al. (2000)
ALDOA	Aldolase, fructose-bisphosphate A	Known exosome protein originated from conceptus and found within uterine fluid of cows and play a role in embryo endometrial interactions during embryo implantation. Increased in Ishikawa cells treated with trophectoderm derived EVs and known to be responsible for Neutrophil activation and immune response	Nakamura et al. (2016), Poh et al. (2021)
NASP	Nuclear autoantigenic sperm protein	Shown as required for bovine pre-implantation embryonic development and is associated with cell proliferation, DNA replication	Nagatomo et al. (2016)
PDIA4	Protein disulfide isomerase family A member 4	PDI is known to regulate expression of implantation related molecules and PDI4 is specifically altered in receptive endometrium and plays a role in embryo attachment	Fernando et al. (2021)
PPIA	Peptidylprolyl isomerase A	PPIA is found in uterine fluid EVs isolated from fertile female and known to be associated with success of implantation	Rai et al. (2021)
PDIA3	Protein disulfide-isomerase A3	PDIA3 is known to regulate the proliferation and apoptosis through MDM2/p53 pathway	Mo et al. (2020)
BASP1	Brain acid soluble protein 1	Epithelial loss of progesterone receptor (PGR) resulted in downregulation of BASP1 along with impaired progesterone (P4) signalling, altered cell differentiation, and disrupted signalling pathways, leading to embryo implantation failure	Gebril et al. (2020)

## 4 Discussion

Both 3D and 2D cultures are recognized as having applications in cancer research, drug discovery and in understanding physiological and pathological processes within biological systems. Three dimensional cultures are becoming more attractive in the field of drug discovery and tissue engineering owing to their ability to better mimic the *in vivo* cellular microenvironment. While the majority of EV studies have been conducted using EVs from 2D cell cultures, there is evidence suggesting that 2D and 3D cell secretomes and EV compositions differ. With the increasing interest in EVs as a therapeutic modality to improve the embryo implantation process, standardized production of EVs remains a challenge. While 3D cultures offer opportunities to scale up EV production, how the cellular micro-environment could affect EV production, bioactivity, and molecular composition remain elusive. Therefore, we studied whether the composition of EVs derived from cells grown in 3D and 2D microenvironment is different and how this affects the EV mediated cellular communication using an embryo implantation model. In the current study, we show that EV signalling can be tuned based on the proteomic composition of the EVs, which seems to depend on the cellular microenvironment where the donor cells were grown.

There are limited availability in primary tissues to isolate fresh trophoblast cells, therefore cell line derived from normal placenta, embryonal carcinomas and malignant tissue with evidence of trophoblast differentiation are imperative for EV production and functional studies. Some cell lines used as mimics of trophoblast cells are JAR, BeWo, and JEG-3, and these cell lines produce hormones and show cell growth similar to normal trophoblast cells (Grummer et al., 1994). These cell lines can be far from perfect, however, they can be used to generate significant interventional targets for clinics that can be later validated for more appropriate source materials or used for EV engineering purposes. Additionally, obtaining enough material for EV isolation from cell culture is challenging. EV studies typically employ three cell culture techniques, which includes, monolayer 2D culture, scaffold-based 3D culture, and scaffold-free 3D culture (Langhans, 2018; Chen and Wang, 2020). EV production can be scaled up using cell growth in bioreactors, induction of EV secretion from cells using stressors (by modulation of culture conditions, or by using physical and chemical stimulants) and by cell fragmentation methods (Syromiatnikova et al., 2022). The choice of cell culture method mostly depend on the specific application. In 2D monolayer cultures, cells grow flat in shape and are in contact with their neighbouring cells, cell culture media, and the culture vessel. However, they lack spatial polarization and cell-extracellular matrix (ECM) interactions (Kapałczyńska et al., 2016). In scaffold based 3D cultures, cells are basically in contact with the neighbouring cells, scaffold and cell culture media. In scaffold-free 3D cultures, cells are in contact with neighbouring cells and the culture medium. Non-scaffold-based 3D cultures are suspension cultures grown non-adherently to the culture surface such as hanging drop cultures, magnetic levitation and rotary cell cultures. Scaffold-based 3D cultures have concerns regarding biocompatibility and utility in clinical applications as they use non-human material. The cells in scaffold free culture self-assemble and secrete their own ECM, therefore allow more accurate cell-cell interactions, special organization and physiological responses (Vu et al., 2021). In general 3D culture can be easily scaled up for the large production of EVs, and rotary-shaker base suspension culture is an easy way of producing trophoblast spheroids for EV isolation (Huh et al., 2011; Syromiatnikova et al., 2022).

In the current study, we isolated EVs from trophoblast CM, comparing cells grown as a monolayer and spheroids from rotary shaker-based suspension culture. EVs can be isolated from source materials using different EV isolation techniques such as SEC, ultracentrifugation (UC), filtration, and precipitation methods (Welsh et al., 2024). Methods such as filtration and precipitation result in more protein contaminants and non EV particles in EV preparations. Therefore, the SEC and UC are common methods of EV isolation and can separate EVs from non EV particles and proteins to greater extent. Emerging evidence suggest that EVs produced by SEC has more intact biophysical properties and higher functionality compared to EVs from UC (Mol et al., 2017), therefore we used SEC to isolate EVs. Then, the physical and biochemical properties of the 3D and 2D EVs were characterized according to the guidelines of the Minimal information of Studies of EVs (Théry et al., 2018; Welsh et al., 2024). The secretion dynamics of EVs secreted by 2D vs 3D cells showed that 3D cells secreted more EVs compared to 2D cells, when the similar number cells and time in culture were used for producing EVs. The observed discrepancy in EV production rates between the two culture systems may stem from differences in cell proliferation or how cells are distributed within each culture model. These findings are consistent with the previous reports that suggested active secretion of EVs from 3D culture compared to 2D culture (Villasante et al., 2016; Rocha et al., 2019; Sun et al., 2023). This suggest the possibility of scaling up 3D cell culture for standardized and effective EV production (Guerreiro et al., 2018). While studying real-time EV secretion over extended periods could offer a more comprehensive understanding of EV production efficiency, current technological limitations hinder such investigations. Moreover, spheroids in culture for extended periods of time may start to develop a necrotic core that pose the risk of dying cells releasing EVs mainly consisting of apoptotic bodies rather than exosomes or microvesicles (Abdollahi, 2021). Majority of the EVs derived from both culture systems fell within the size range of 100–200 nm that is typical for small EVs, and TEM images showed that the EVs had a typical cup-shaped morphology. There were slight differences in the size of the particles derived from 2D culture vs 3D culture, where 3D-derived particles were slightly larger than the 2D derived particles. Previous reports also suggest that there can be size differences in EVs of 2D and 3D cell origin when measured with NTA (Thippabhotla et al., 2019; Clément et al., 2022). NTA used to measure the EV size and concentration is based on the principle of light scattering and Brownian motion; however, it is incapable of avoiding any non EV particles from their analysis. Techniques to more specifically label EVs based on antibodies or lipid membrane dyes have been emerged during recent years. To differentiate between EVs and non-EV particles, EVs can be labelled with lipid membrane dyes and then detected under fluorescent mode of the NTA (Midekessa et al., 2021). In contrast to the NTA results, fluorescent NTA showed that the size of the nanoparticles of EV origin derived from both 2D and 3D cells were not different. However, it is also important to keep in mind that this techniques can introduce labelling artefacts leading to over or underestimating the real EV numbers (Welsh et al., 2024). The zeta potential of fluorescent-labelled nanoparticles were different between 2D and 3D EVs, where 3D EVs showed a more negative charge. ZP is one of the most useful tool to investigate the colloidal stability of the nanoparticles, and this characteristics is important in nanoparticle stability and in understanding their pharmacokinetics while preparing nanomedicines (Midekessa et al., 2020). The EVs generated from 3D cells seem more stable compared to 2D EVs, further encouraging their suitability as an EV bio-manufacturing platform. Both 2D EVs and 3D EVs were enriched with classical EV protein markers such as CD9, CD81, CD63, and ITGB. Altogether, this confirmed that EVs could be isolated from both culture conditions and both EV types were of a typical cup shaped with similar mean size range and enriched with classical EV markers.

We next examined the effect of cell culture growth conditions on EV protein cargo composition. The cell growth conditions impacted the proteomic composition of EV cargo. Overall protein secretion was more active in 3D culture compared to 2D culture (more proteins identified in 3D EVs compared to 2D EVs), which was consistent with previous findings (Kusuma et al., 2022). The 2D and 3D EV proteome were clearly distinguishable, with around 153 proteins differentially expressed in 3D EVs compared to 2D EVs. Among the proteins identified, classical EVs proteins were also found. Both the 3D and 2D EV proteomic profiles shared proteins with those previously identified in human trophectoderm stem cell-derived EVs, indicating that the samples were indeed enriched with EVs. Among the differentially expressed proteins, 11 proteins were among the top most common EV proteins, suggesting that, EV cargo protein loading was different in 3D EVs compared to 2D EVs. Interestingly, among these proteins ANXA2 (Garrido-Gómez et al., 2012; Wang and Shao, 2020), SLC3A2 (Poh et al., 2021), and RAC1 (Tu et al., 2016) were identified as critical for embryo adhesiveness to the human endometrium. The key processes of proteins specially enriched in 3D EVs compared to 2D EVs were involved in cell-cell adhesion, cell migration, ECM receptor interaction, regulation of actin-cytoskeleton and the Ras signalling pathway among others. These pathways were previously highlighted as enriched in 3D culture derived EVs (Kusuma et al., 2022). There is no clear consensus on the exact mechanisms of EV secretion, however, cytoskeleton components play important role in EV secretion (Holliday et al., 2019). EV formation and release from a cell involve changes in actin polymerization followed by changes in contraction proteins, and fusion machineries (Rädler et al., 2023). Therefore, we can postulate that morphological changes in cells in spheroid form compared to a monolayer might affect cytoskeleton proteins, leading to changes in EV protein cargo as well. The characteristics of the cellular microenvironment can vary based on several factors, including the properties of the extracellular matrix, the nature and physical properties of the cells themselves (whether they are of the same origin or not), the presence of bioactive molecules like chemokines, cytokines and growth factors secreted by cells, and mechanical forces exerted by the surrounding fluid (Barthes et al., 2014). Consequently, cells cultivated in 2D and 3D environments are expected to exhibit different physiochemical properties. For instance, in 3D rotary culture, the movement of cell culture media generates variations in fluid shear stress on cells compared to the static environment of 2D culture. Fluid shear stress can be transmitted to the cell plasma membrane, potentially influencing tension at cell-cell junctions and subsequently altering the proteins associated with these junctions (White and Frangos, 2007). The spheroid formation take few steps. In the first step cadherin-cadherin and integrin binding to ECM form a loose cellular aggregate which is further compacted over time. After the cellular re-organization period cell aggregate morphologically transit in to spheroids and this is mainly mediated by cadherins (Egger et al., 2018). Interestingly the trophoblast EVs generated in 3D culture compared to 2D culture also seems to reflect these changes as shown in the pathway analysis.

The functional effects of 2D and 3D EVs were assessed in relation to EV-mediated embryo-maternal communication. We previously identified that the MFGE8 protein secretion increases in receptive endometrial epithelial cells in response to trophoblast-derived EVs, suggesting it as a potential marker of EV mediated intercellular communication between the embryo and the endometrium. To gain insight into the effects of molecular cargo differences on EV signalling, we assessed MFGE8 secretion from EEC in response to 2D and 3D EVs, respectively. MFGE8 secretion was increased in EECs treated with both 2D and 3D EVs. Nevertheless, the data suggested that there are distinct differences in the activity of 2D and 3D EVs in tuning MFGE8 secretion from endometrial cells. Interestingly, the effects of 2D EVs on the increase in MFGE8 secretion was higher than that of 3D EVs showing differences in EV potency. Increased potency of 2D EVs compared to 3D EVs has been reported previously in mesenchymal stem cell derived EVs as well (Sun et al., 2023). It can be postulated that the tuning of paracrine EV signalling depends up on their parent cell characteristics and microenvironment in which they were grown. MFGE8 protein expression is required for embryo attachment (Schmitz et al., 2014). Additionally, MFGE8 can influence endometrial stromal cell apoptosis, thereby facilitating embryo invasion (Riggs et al., 2012). Therefore it can be postulated that 2D and 3D EVs may have different potencies in mediating embryo implantation process. Previous literature suggest that molecular changes between 3D and 2D EV preparations may be implicated in other cellular processes such as the immunological processes, extracellular matrix or membrane re-organization or cell proliferation and migration processes (Thippabhotla et al., 2019). However, there are also instances where the molecular cargo differences between 2D and 3D EVs do not translate into functional disparities (Kusuma et al., 2022). So more studies are required in this context for different cell types and culture conditions.

Finally, the 2D and 3D EV protein cargo composition was compared with secretions from the single human embryo prior to implantation and with known endometrial receptivity markers. The majority of those proteins common to trophoblast EVs and human embryo secretions were implicated to be involved in the processes of embryo development, endometrial receptivity, and implantation suggesting the potential value of trophoblastic EVs as a therapeutic tool to facilitate embryo implantation process. Trophoblast cells undergo a transformation into a significant portion of the placenta, an essential fetal organ that facilitates the connection between the fetus and the maternal bloodstream. Within this structure, they release a substantial number of vesicles into the maternal bloodstream (Tong and Chamley, 2015). This vesicle transfer plays a crucial role in facilitating feto-maternal communication, aiding in fetal development, mediating maternal immune tolerance, and sustaining the pregnancy (Buca et al., 2020; Martin et al., 2022).

Less standardized protocols for EV production hinders progress in their clinical translation. Although 3D culture is recognized for better mimicking the *in vivo* cellular status compared to 2D culture, there remains a gap in understanding EV characteristics, composition, and signalling in relation to cell growth conditions. According to previous reports multiple factors can affect EV production from cells. For example, mesenchymal stem cells cultured in both 2D and 3D environments cell culture conditions exhibited variations in cell secretome and EVs, thereby impacting cellular functionality (Carter et al., 2019; Kusuma et al., 2022). Additionally, cell culture media composition (such as presence of FBS, EV depleted FBS or serum starved media) may also affect the EV production and their characteristics (Lehrich et al., 2021). Interestingly, bidirectional fluidic flow in primary liver cell cultures has been shown to alter cellular metabolic activity significantly compared to 2D static culture, potentially due shear stress sensing, increased metabolic and gas exchange, or through accumulation of growth factors (Esch et al., 2015). Therefore, it is crucial to consider factors such as culture status (static or flow culture) or composition, fluid flow rate, fluid flow direction and pattern of cell-matrix interactions when producing EVs, as these can impact not only the cells but also the EVs they generate, leading to changes in cellular signalling. In conclusion, this study provides compelling evidence of how changes in the cellular microenvironment are reflected in the protein cargo of EVs, subsequently affecting EV-mediated intercellular communication. Nevertheless, the EV characteristics, composition and potency may also vary depending on the cell type from which they originate. Therefore, it would be valuable to investigate these aspects in primary trophoblast cells or other cell lines of trophoblastic origin. Future studies should focus on investigating the potential effects of each of the above factors on EV production in different culture systems to establish standardized protocols for EV production for translational or other purposes.

## 5 Conclusion

In conclusion, the role of EVs in embryo maternal communication is beginning to be unveiled, opening new avenues for the development of diagnostics and therapeutics. However, this is largely limited by a lack of standardized bio-manufacturing methods for EV mass production. While 3D cultures offers opportunities to scale-up EV production, how the cellular microenvironment could affect EV production, bioactivity and molecular composition remained elusive. Our comparison of EVs generated by 2D and 3D cells showed a remarkable variation between the trophoblast EVs cargo proteome and functionality within the embryo implantation model. The findings suggest that there is an effect of cellular architecture on EVs generated from them, which then probably has an effect on the potency of EV signalling at the recipient cell level. As a whole, caution is warranted when selecting an EV manufacturing platform, especially for assessing the functional and therapeutic potential of EVs.

## Data Availability

The data presented in this study are available in the ProteomeXchange Consortium via the PRIDE with the dataset identifier PXD048789.
