# Supplementary material for: Effect of 3D and 2D cell culture systems on trophoblast extracellular vesicle physico-chemical characteristics and potency
Source: Front Cell Dev Biol. 2024 May 21;12:1382552. doi: 10.3389/fcell.2024.1382552 (PMC11148233; doi:10.3389/fcell.2024.1382552)
Supplement: Supplementary file 2 [file Table2.DOCX]

Supplementary figure 1


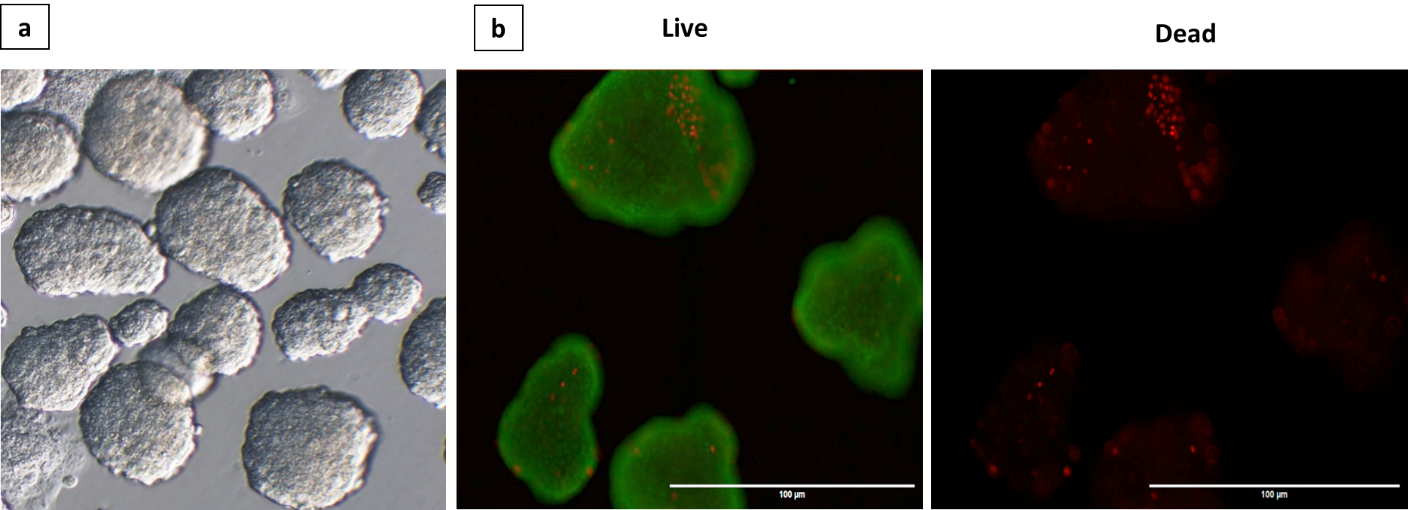


JAr spheroids (3D model). (a) Light microscopy images showing 3D JAr spheroids after 48 h of incubation. (b) Fluorescent microscopy images showing cell viability of spheroids, viable cells appear as green, while nonviable cells appear as red.
